# Fecal *Enterotoxigenic Bacteroides fragilis*–*Peptostreptococcus stomatis*–*Parvimonas micra* Biomarker for Noninvasive Diagnosis and Prognosis of Colorectal Laterally Spreading Tumor

**DOI:** 10.3389/fonc.2021.661048

**Published:** 2021-05-11

**Authors:** Xiaonan Shen, Jialu Li, Jiaqi Li, Yao Zhang, Xiaobo Li, Yun Cui, Qinyan Gao, Xiaoyu Chen, Yingxuan Chen, Jing-Yuan Fang

**Affiliations:** Key Laboratory of Gastroenterology and Hepatology, Ministry of Health, Division of Gastroenterology and Hepatology, Shanghai Institute of Digestive Disease, Renji Hospital, School of Medicine, Shanghai Jiaotong University, Shanghai, China

**Keywords:** laterally spreading tumor, *enterotoxigenic Bacteroides fragilis*, *Peptostreptococcus stomatis*, *Parvimonas micra*, noninvasive biomarker, diagnosis and prognosis

## Abstract

**Objective:**

Up to now, non-invasive diagnosis of laterally spreading tumor (LST) and prediction of adenoma recurrence after endoscopic resection of LSTs is inevitable. This study aimed to identify a microbial signature with clinical significance of diagnosing LSTs and predicting adenoma recurrence after LSTs colectomy.

**Methods:**

We performed 16S rRNA sequencing in 24 mucosal samples, including 5 healthy controls (HC), 8 colorectal adenoma (CRA) patients, and 11 LST patients. The differentiating microbiota in fecal samples was quantified by qPCR in 475 cases with 113 HC, 208 CRA patients, 109 LST patients, and 45 colorectal cancer (CRC) patients. We identified differentially abundant taxa among cases and controls using linear discriminant analysis effect size analysis. ROC curve was used to evaluate diagnostic values of the bacterial candidates. Pairwise comparison of AUCs was performed by using the Delong’s test. The Mantel-Haenszel hazard models were performed to determine the effects of microbial compositions on recurrence free survival.

**Results:**

The microbial dysbiosis of LST was characterized by relative high abundance of the genus Lactobacillus-Streptococcus and the species enterotoxigenic Bacteroides fragilis (ETBF)–Peptostreptococcus stomatis (P. stomatis)–Parvimonas micra (P. micra). The abundance of ETBF, P. stomatis, and P. micra were steadily increasing in LST and CRC groups. P. stomatis behaved stronger value on diagnosing LST than the other two bacteria (AUC 0.887, 95% CI 0.842–0.931). The combination of P. stomatis, P. micra, and ETBF (AUC 0.922, 95% CI 0.887–0.958) revealed strongest diagnostic power with 88.7% sensitivity and 81.4% specificity. ETBF, P. stomatis, and P. micra were associated with malignant LST (P_P.stomatis_ = 0.0015, P_P.micra_ = 0.0255, P_ETBF_ = 0.0169) and the abundance of IL-6. The high abundance of P. stomatis was related to the adenoma recurrence after LST resection (HR = 3.88, P = 0.008).

**Conclusions:**

Fecal microbiome signature (*ETBF*–*P. stomatis*–*P. micra*) can diagnose LSTs with high accuracy. *ETBF*, *P. stomatis*, and *P. micra* were related to malignant LST and *P. stomatis* exhibited high predictive value on the adenoma recurrence after resection of LSTs. The fecal microbiome signature of LST may provide a noninvasive alternative to early detect LST and predict the adenoma recurrence risk after resections of LSTs.

## Introduction

Globally, colorectal cancers (CRC) ranked second in mortality and third in incidence among cancers in 2020 (1). Nearly 90% of colorectal cancers develop in the adenoma-carcinoma sequence over 10 years (2, 3). Adenomas are crucial precursor lesions of CRC. To avoid the carcinogenesis progress, adenomas can be removed by colonoscopic resection. Laterally spreading tumors (LSTs) are well known as primary precursor lesions of CRC (4, 5). LSTs are featured by horizontally extending growth patterns and are at least 1cm in diameter (6). It is classified into two types: LST-Gs with granules or nodules on surfaces and LST-NGs featured with smooth surfaces without nodules or granules (6). LST-Gs are further categorized into nodular mixed (LST-G-M) and homogeneous (LST-G-H) subtypes. LST-NGs include pseudo-depressed (LST-NG-PD) and flat-elevated (LST-NG-FE) subtypes. The detection of LST is extremely difficult due to the special morphology and growth pattern. Thus, there is a need to sensitively detect LSTs to prevent the incidence of CRC. Moreover, adenoma recurrence after endoscopic resection of LSTs is frequent (7, 8). Despite the widespread use of endoscopic resection, few studies examined the risk factors for adenoma recurrence after LST resection. Many LST patients without adenoma recurrence were subjected to ultimately and frequently redundant surveillance procedures. Hence, a non-invasive, economic and convenient marker that can sensitively detect LSTs and predict the adenoma recurrence of LSTs yields to be explored.

The colorectal epithelium has a constant crosstalk with approximately 3 × 10^13^ gut microorganisms (9). In the past 5 years, the roles of gut microbiota in the carcinogenesis of CRC have received lots of attention. Human studies generally showed that the gut microbiota related to CRC patients was significantly different compared with healthy individuals. It showed higher species richness and increased abundant procarcinogenic taxa (such as *Fusobacterium, Bacteroides, Porphyromonas*, *Escherichia*) and lower abundance of potentially protective taxa (10–12). And these studies pinpoint a potential core series of carcinogenic microorganisms and provide scientific resource for developing fecal microbial indicators for the diagnosis of CRC. Existing studies have applied the abundance of multiple fungal, bacterial, viral species to differentiate CRC patients from HC and these noninvasive biomarkers exerted high sensitivity and specificity (12–17). Apart from microbial diagnostic applications, relations concerning clinical outcomes of CRC and bacterial biomarkers have raised the probability of prognostic bacterial markers. Numerous studies have disclosed the associations between CRC survival and tumoral bacterial abundance (18–20). The microbiota may be an effective, noninvasive, economic, and convenient biomarker for prognosis of CRC.

LST, an important precursor of CRC, was hardly recognized and resected for flat morphology during endoscopy. Exploring the microbiome signature of LSTs may inspire new approaches to identify or slow LSTs progressions. Current noninvasive screening tests including tumor markers and fecal occult blood test have low sensitivities for detecting LST. While microbiome signature for LSTs and noninvasive stool-based screening tests remain rare-reported. For widely screen and surveillance of LSTs, cost-effective, non-invasive biomarkers are urgently required, which are apparently beneficial to patients’ compliance.

In this study, we identified a microbiome signature with clinical significance to detect LSTs from healthy controls (HC) and colorectal adenoma (CRA). And we identified the microbiota with high accuracy on predicting the adenoma recurrence after colonoscopic resection of LSTs.

## Materials and Methods

### Study Design and Participants

**Figure 1:**
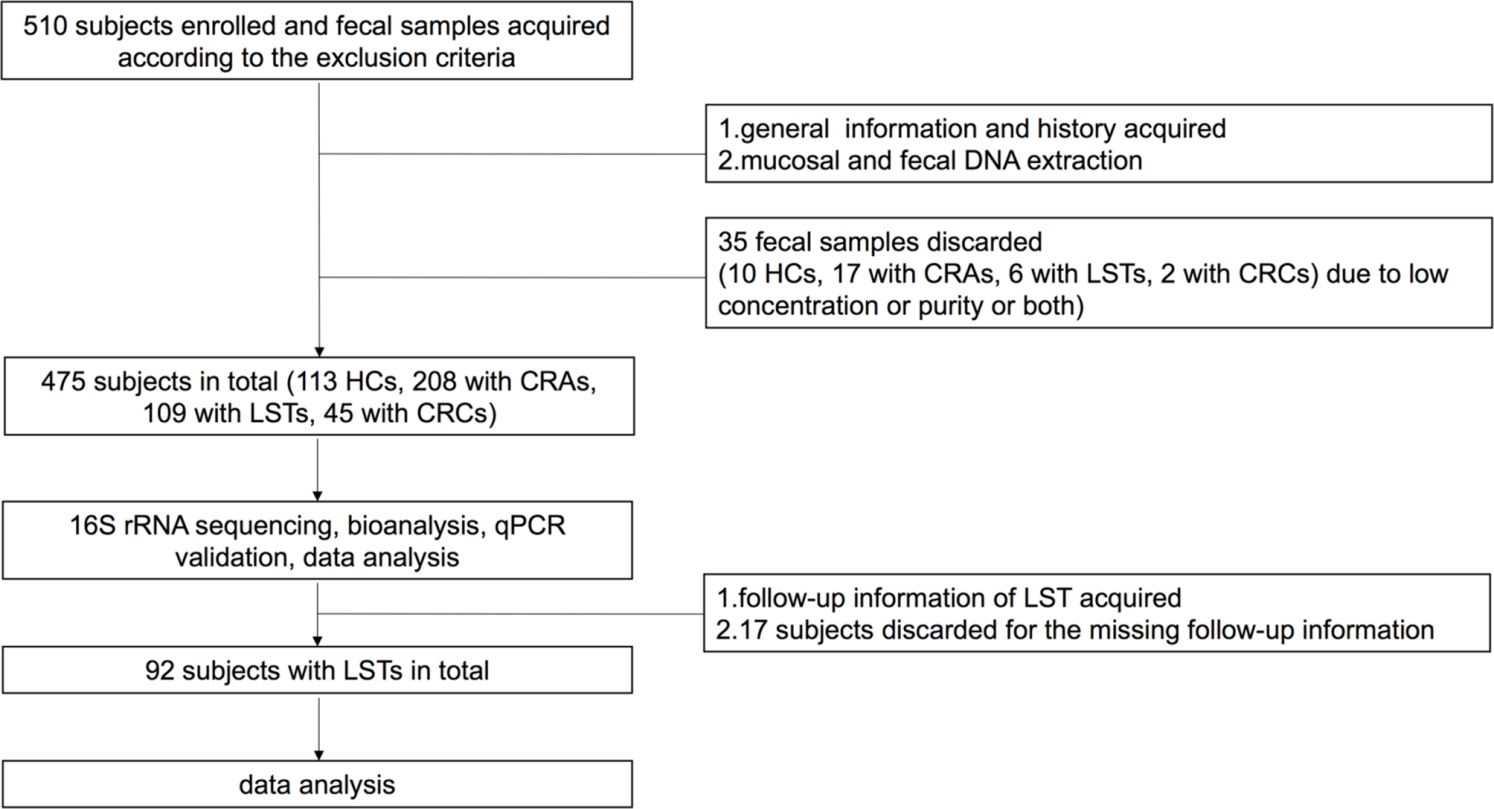
Work flowcharts. HC, healthy control; CRA, colorectal adenoma; LST, laterally spreading tumor; CRC, colorectal carcinoma; qPCR, quantitative polymerase chain reaction.

This retrospective cohort study was carried out at Renji Hospital, School of Medicine, Shanghai Jiaotong University and approval for the study was obtained from the ethics committee of Renji Hospital, School of Medicine, Shanghai Jiaotong University. The study complied with the Helsinki Declaration of 1975. A total number of 475 patients was consisted with 113 HCs, 208 CRA patients, 109 LST patients, and 45 CRC patients (work flowchart see **Figure 1**). Excluding criteria were established to prevent potential gut microbial alternation. The exclusion criteria included: 1) with the upper gastrointestinal (GI) tracts surgery history; 2) with the history of hereditary non-polyposis CRC (Lynch syndrome), familial adenomatous polyposis (FAP), or Peutz-Jeghers syndrome or uncontrolled chronic metabolic disorder, including diabetes and hypertension; 3)with uninterested GI tract neoplasia history; 4) active GI tracts bleeding in recent six months; 5) using probiotics, antibiotics, immunosuppressor, nonsteroidal anti-inflammatory drugs at least 1 month before enrollment; 6)with eating habits changes in recent 1 month (17).

Enrolled subjects were divided into four groups: HC group, CRA group, LST group and CRC group and the clinical phenotype was defined by pathological and endoscopic diagnosis. Distal tumors were considered to be those in descending colon, sigmoid colon and rectum, while proximal tumors included those in the rest of colon. Conventional-type CRA include villous adenoma, tubular adenoma, tubulovillous adenoma. Serrated polyps include hyperplastic polyps and serrated adenoma. The early stage CRC (S0) as well as stage 0/pTis CRC was defined as adenoma with high-grade dysplasia and intramucosal carcinoma. Adenoma recurrence was endoscopically confirmed under surveillance in 3 to 36 months and was defined as detection of recurrent adenomas in patients without the recurrence evidence during any previous surveillance period. Late adenoma recurrence was described as recurrent adenoma at the site of previous resection at ≥12 months’ surveillance.

### Stool and Mucosal Samples Collection

Before bowel preparation for endoscopy, all 475 stool samples (113 HC, 208 CRA patients, 109 LST patients, 45 CRC patients) were collected in the germ-free containments. All stool samples were temporarily preserved in −20°C and transferred to −80°C for storage within 48 h. All 24 mucosal samples (5 HC, 8 CRA patients, 11 LST patients) were collected in the special germ-free containment after endoscopic resection and were immediately moved to −80°C for storage.

### DNA Extraction, 16S rRNA Gene Sequencing, and Quantitative Polymerase Chain Reaction

QIAamp DNA Tissue Mini Kit and QIAamp DNA Stool Mini Kit were used according to the instructions (Qiagen, Hilden, Germany). All extracts were preserved at −80°C before 16S rRNA sequencing and subsequent polymerase chain reaction (qPCR). We performed 16S rRNA sequencing in mucosal samples. The 16S rRNA V3-V4 region was sequenced on the Miseq platform (Illumia, San Diego, California, USA). We verify the potential microbiota by qPCR according to the sequencing results. The relative primers sequences were described in **Supplementary Table 1**. All SYBR-green based qPCR samples were in triplicates and average Ct value was calculated. The relative abundance of the target gut microbiota was based on the ΔCt value defined as the target Ct value subtracted Ct value for 16s rRNA (21, 22). 10 μl SYBR Green II was the qPCR reaction system using SYBR^®^Premix Ex TaqTMII (TliRNaseH Plus) of TAKARA cooperation. Stepone^®^plus by ABI company was used in all operations and configurations in 40 cycles of 95°C denaturation for 5 s, 60°C annealing and 30 s extension after pre-denaturation at 95°C for 30 s.

### Statistical Analysis

The 16S rRNA sequencing statistics were analyzed by Quantitative Insights Into Microbial Ecology (QIIME2 V.2018.2). Basic bioinformatics analysis included reads splicing quality control and OTU clustering analysis. Reads splicing quality control is the original sequencing data using QIIME to filter the sequence quality according to the following criteria: 1) The two-terminal sequences obtained by MiSeq sequencing were spliced into one sequence. 2) The minimum overlap length is 10 bp and the maximum mismatch ratio allowed in the overlap region is 0.2. 3) Barcode, the sequence used to distinguish different samples, required the exact match. The maximum number of mismatches for the primer is 2. OTU was applied according to the similarity of the sequence. The specific OTU clustering steps were as following: 1) extracting the non-repetitive sequence in the optimized sequence; 2) performing OTU clustering under the criterion of 97% similarity. The OTU information have been uploaded on. We used the Bayesian algorithm to classify the OTU representative sequence in the domain, kingdom, phylum, class, order, genus, and species levels referring to the Silva database (http://www.arb-silva.de). To investigate whether the sample size of discovery cohort was sufficient, we performed Core curve analysis and Rarefaction curve analysis. With the sample size increasing, the Core curve tended to be horizontal, which indicated the number of core species did not change significantly. In Rarefaction curve analysis, as the number of sequencing samples increasing, the Shannon diversity indices tended to be flat with increasing number of sequencing samples. Rarefaction curve analysis indicated that the sequencing data were reasonable as well. The Core curve analysis and Rarefaction curve of mucosal samples indicated that our sample size was suitable for 16S rRNA sequencing (**Supplementary Figure 1**). Then we performed the entero-typing analysis on family, genus, and species levels and performed the distance heatmap, heatmap, cicros, and tertiary analysis to find the differentially taxa among groups. We identified differentially abundant species among cases and controls using linear discriminant analysis effect size (LEfSe) analysis. To explore the relationship between the targeted bacteria and other bacteria, we use the GMrepo bacteria database (https://gmrepo.humangut.info). The other statistical analysis involved Kruskal-Wallis test, Fisher’s exact test and partial Spearman’s rank correlation (PResiduals package).

All qPCR samples were in triplicates. All the relative abundance was normalized by the abundance of 16S rRNA. Average Ct value was calculated from the triplicates. The ΔCt value was defined as the target microbioma Ct value subtracted Ct value for 16S rRNA and the relative abundance of the target microbiota was based on ΔCt (21). Kruskal-Wallis test and Mann-Whitney U test were respectively used in comparison for nonparametric and continuous analysis. Receiver operating characteristic (ROC) curve evaluated the diagnostic value of bacterial candidates in differentiating LST from HC and CRA. And the ROC curve was used to establish the cut-off value maximizing the Youden index (J = Sensitivity + Specificity − 1). The Delong’s test was performed to pairwisely compare areas under ROC (AUCs). Recurrence-free survival (RFS) was described as the time from diagnosis to the colorectal adenoma recurrence in 36 months. Patients without adenoma recurrence were censored at date of last surveillance. Mantel-Haenszel hazard models determined the effects of microbial composition on RFS. All tests were done by SPSS statistical software (version 23.0, IBM Corp, Armonk, New York, 2012) and R statistical software (version 3.6.0, 2019).

## Results

### Bacteria Composition Was Significantly Different Between LSTs and Controls

**Figure 2:**
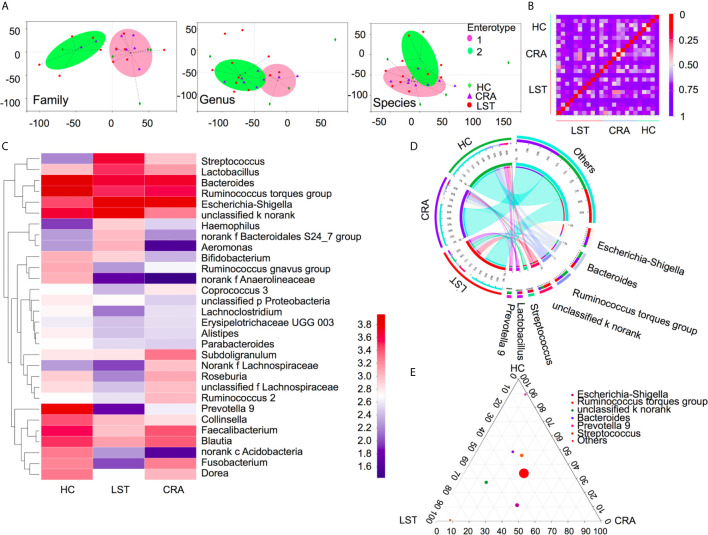
Variations of mucosal microbiota composition in LST. **(A)** Entero-typing analysis on family, genus and species groups in the HC (*n* = 5), CRA (*n* = 8), and LST group (*n* = 11). The green diamond represented the HC group. The purple triangle represented the CRA group and the red dot represented the LST group. The pink dot represented the enterotype 1 while the green dot represented the enterotype 2. **(B)** Distance heatmap in samples on genus level. **(C)** Heatmap in the three groups on genus level. **(D)** Cicros analysis on genus in three groups. **(E)** Tertiary analysis on genus level in three groups.

**Figure 3:**
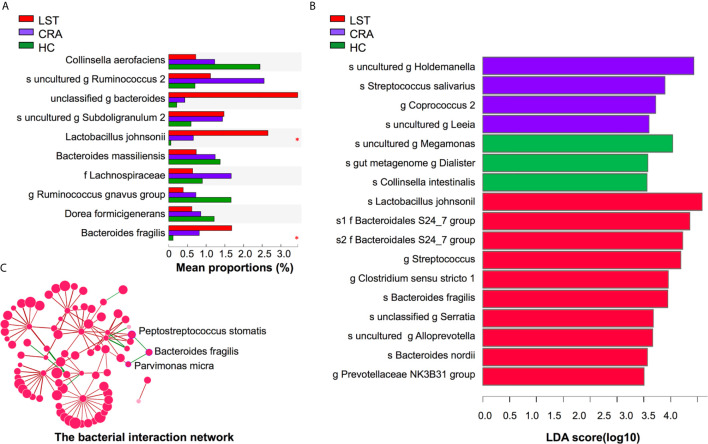
Variations of mucosal microbiota composition on species level in LST. **(A)** Kruskal-Wallis test analysis on species level in the three groups. The X axis represented the mean proportions (%) of species in three groups. *Lactobacillus johnsonii* and *Bacteroides fragilis* presented high abundance in the LST group. **(B)** LDA analysis on species level in the three groups. The X axis represented LDA score of species in three groups. *Lactobacillus johnsonii* and *Bacteroides fragilis* significantly over-presented in the LST group. **(C)** The interaction network among *Bacteroides fragilis* and other bacteria. The abundance of *Bacteroides fragilis* was positively related with the abundance of *Peptostreptococcus stomatis* and *Parvimonas micra*. LDA, linear discriminant analysis; HC, healthy control; CRA, colorectal adenoma; LST, laterally spreading tumor. *P < 0.05.

A total number of 475 patients were divided into 113 HCs, 208 CRA patients, 109 LST patients, and 45 CRC patients (basic demographic features see **Supplementary Table 2**; Work flow chart see **Figure 1**). To examine the bacteria composition in LSTs, the 16S rRNA sequencing was performed in 24 mucosal samples (5 HCs, 8 CRA patients, 11 LST patients). Bacteria compositions in HCs, CRA patients, and LST patients were compared. In entero-typing analysis, it was found that bacteria were significantly different in family, genus, and species levels among the above three groups (**Figure 2A**), as well as in the distance heatmap analysis on genus level (**Figure 2B**). *Lactobacsillus* and *Streptococcus* were abundant on genus level in LST group compared to the other two groups (**Figures 2C–E**, **Supplementary Figure 2**). The potential differential bacterium patterns among the three groups was defined by Kruskal-Wallis test and LEfSe algorithm. *Lactobacillus johnsonil* (*L. johnsonil*) and *Bacteroide fragilis* were significantly abundant in LST group (**Figures 3A, B** **)**. By analyzing the GMrepo bacteria database, 19 bacteria co-occurred with *Bacteroide fragilis* in colorectal neoplasms. We found only two bacteria showed the positive correction coefficient in both Pearson’s and Spearman’s analyses (**Supplementary Table 3**). The abundance of *Peptostreptococcus stomatis* (*P. stomatis*) and *Parvimonas micra* (*P. micra*) were found to be positively relevant to the abundance of *Bacteroide fragilis* (**Figure 3C**). Moreover, *enterotoxigenic Bacteroides fragilis* (*ETBF*) was reported to be driver bacteria in colorectal cancer development (23–27). Thus, we focused on verifying the abundance of *ETBF*, *P. stomatis*, *P. micra*, and *L. johnsonil* in cases.

### The Abundance of *ETBF, P. stomatis,* and *P. micra* were Increased in LST and CRC Groups

**Figure 4:**
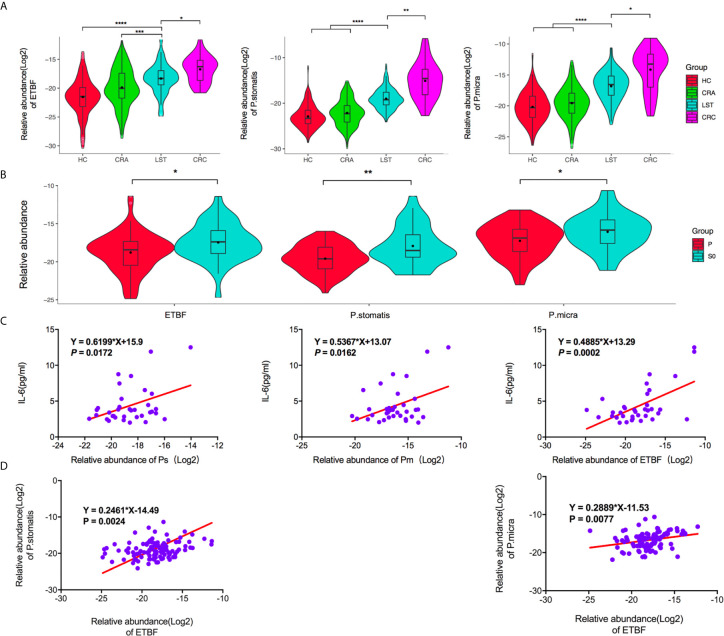
The fecal relative abundance of *ETBF, P. stomatis*, and *P. micra* among groups. **(A)** The fecal relative abundance of *ETBF, P. stomatis*, and *P. micra* was stepwise increased in the three groups. **(B)** The fecal high abundance of *ETBF, P. stomatis* and *P. micra* was relevant with S0 stage LSTs. **(C)** The expression of IL-6 was positively related with the abundance of the three bacteria. **(D)** The abundance of *P. stomatis* and *P. micra* increased with the increasing abundance of *ETBF*. HC, healthy control; CRA, colorectal adenoma; LST, laterally spreading tumor; CRC, colorectal carcinoma; *ETBF, Enterotoxigenic Bacteroides fragilis; P. stomatis, Peptostreptococcus stomatis; P. micra, Parvimonas micra;* P, LSTs with hyperplastic type or low-grade dysplasia; S0, LSTs with intramucosal carcinoma or high-grade dysplasia. *P < 0.05. **P < 0.01. ***P < 0.001. ****P < 0.0001.

*ETBF, P. stomatis, P. micra,* and *Lactobacillus johnsonil* were also tested in fecal samples. Increased abundance of *ETBF, P. stomatis*, and *P. micra* in LST patients compared with HCs and CRA patients was observed. Moreover, there was an increase of the above microbiota in CRC patients over LST patients, as well (**Figure 4A**). Comparing LST patients with HCs, *ETBF* increased by 9.26 folds (*P* < 0.0001), *P. stomatis* increased by 14.79 folds (*P* < 0.0001), and *P. micra* increased by 10.17 folds (*P* < 0.0001). Comparing CRA patients with LST patients, *ETBF* increased by 2.95 folds (*P* < 0.0001), *P. stomatis* increased by 8.76 folds (*P* < 0.0001), and *P. micra* increased by 6.65 folds (*P* < 0.0001). Comparing LST patients with CRC patients, *ETBF* increased by 3.00 folds (*P* = 0.0327), *P. stomatis* increased by 15.31 folds (*P* = 0.0058), and *P. micra* increased by 6.41 folds (*P* = 0.0413) in CRC patients group. Further, no discrepancy was found for the relative abundance of three intestinal bacteria among distal and proximal colon for LSTs (**Supplementary** **Figure 3A**). In terms of topography and histopathology, no distinction was found between LST-G and LST-NG, as well as traditional adenoma type and serrated polyp type (**Supplementary Figures 3B, C** **)**. In addition, the dysplasia degree of adenoma led to totally different prognosis and clinical decisions. Accordingly, the three bacteria in terms of dysplasia degree reported by pathologist were tested. The S0 LST had higher abundance of fecal bacteria (*P_P.stomatis_ =* 0.0015, *P_P.micra_*= 0.0255, *P_ETBF_ =0.0169)* (**Figure 4B**). To further explore the relationship between inflammatory cytokines and the above three bacteria, we collected the clinical laboratory test results of TNF-α, IL-1β, IL-6, IL-8, IL-10 of 35 LST patients (**Supplementary Table 4**). The results showed that *P. stomatis*, *P. micra*, and *ETBF* significantly increased IL-6 secretion (**Figure 4C**) while the secretion of TNF-α, IL-1β, IL-8, IL-10 showed no significant relation with the above three bacteria (**Supplementary Figure 4**). Moreover, the abundance of *P. stomatis* (*P_P.stomatis_ =* 0.0024) and *P. micra* (*P_P.micra_ =*0.0077) positively correlated with the abundance of *ETBF* (**Figure 4D**).

As for *Lactobacillus johnsonil*, no significant changes have been revealed among CRA and LST groups (**Supplementary Figure 5**). Therefore, *ETBF*, *P. stomatis*, and *P. micra* were selected as non-invasive candidate biomarkers for detecting LSTs.

### The Predictive Values of *ETBF*, *P. stomatis*, and *P. micra* for the Occurrence of LSTs

**Figure 5:**
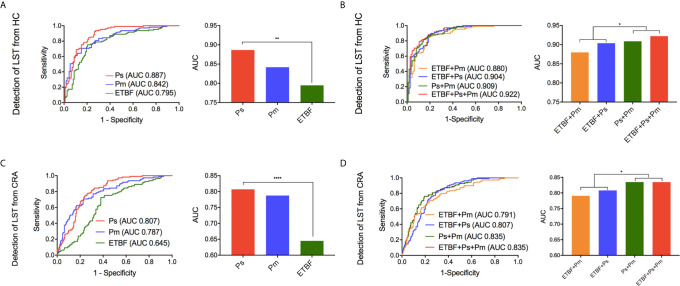
The diagnostic performance of marker *ETBF*, *P. stomatis, P. micra*, and combined test by ROC curve analysis. **(A)** Differentiating LST from HC, the ROC curves and AUCs (Delong’s test) of the three bacteria. **(B)** Differentiating LST from HC, the ROC curves and the AUCs (Delong’s test) of the promising diagnostic bacterial models. **(C)** Differentiating LST from CRA, the ROC curves and AUCs (Delong’s test) of the three bacteria. **(D)** Differentiating LST from CRA, the ROC curves and the AUCs (Delong’s test) of the promising diagnostic bacterial models. HC, healthy control; LST, laterally spreading tumor; *ETBF*, *Enterotoxigenic Bacteroides fragilis; Ps*, *Peptostreptococcus stomatis*; *Pm, Parvimonas micra;* AUC, area under the receiver-operating characteristic curve. *P < 0.05, **P < 0.01, ****P < 0.0001.

The relative abundance of the *ETBF*–*P. stomatis*–*P. micra* microbiome showed an increasing in the LST group as compared to HC group and CRA group as described above (**Figure 4A**). In comparison between HC group and LST group, the ROC curves were performed. In terms of AUCs, *P. stomatis* behaved stronger than the other two bacteria (AUC*_P.stomatis_*0.887, 95% CI 0.842–0.931) (**Figure 5A**). These three contributing markers were therefore united as an independent model. Combination of *P. stomatis*–*P. micra* and *P. stomatis*–*P. micra-ETBF* revealed stronger diagnostic potential over the single *P. stomatis* (*P_P.stomatis_*_–_ *_P.micra_* = 0.0376, *P_P.stomatis_*_–_ *_P.micra-ETBF_*=0.00930)(**Figure 5B**). The combination of the three bacteria showed the maximum AUC (AUC 0.922, 95% CI 0.887–0.958) and Youden index with 88.7% sensitivity and 81.4% specificity (**Table 1**).

**Table 1:** AUC and diagnostic value of different biomarkers for predicting LST.

		AUC	95% CI		Sensitivity	Specificity	Youden Index
**Detection LST from HC**	**ETBF**	0.795	0.735	0.855	0.752	0.797	0.549
	**Ps**	0.887	0.842	0.931	0.936	0.726	0.661
	**Pm**	0.842	0.790	0.893	0.706	0.850	0.556
	**ETBF+Ps**	0.904	0.864	0.944	0.899	0.788	0.687
	**ETBF+Pm**	0.880	0.835	0.925	0.844	0.823	0.667
	**Ps+Pm**	0.909	0.871	0.947	0.862	0.814	0.677
	**ETBF+Ps+Pm**	0.922	0.887	0.958	0.881	0.814	0.695
**Detection LST from CRA**	**ETBF**	0.645	0.584	0.707	0.725	0.620	0.345
	**Ps**	0.807	0.760	0.854	0.844	0.678	0.522
	**Pm**	0.787	0.735	0.840	0.706	0.769	0.476
	**ETBF+Ps**	0.808	0.761	0.855	0.835	0.688	0.522
	**ETBF+Pm**	0.791	0.739	0.842	0.633	0.841	0.474
	**Ps+Pm**	0.835	0.790	0.879	0.762	0.808	0.569
	**ETBF+Ps+Pm**	0.835	0.790	0.879	0.762	0.808	0.569

HC, healthy control; CRA, colorectal adenoma; LST, laterally spreading tumor; ETBF, Enterotoxigenic Bacteroides fragilis; Ps, Peptostreptococcus stomatis; Pm, Parvimonas micra.

To distinguish LSTs from CRAs, the ROC curves were made. The result showed that *P. stomatis* exhibited higher AUCs than the other two bacteria (AUC*_P.stomatis_*0.807, 95% CI 0.760–0.854)(**Figure 5C**). Nonetheless, the diagnostic combination of *P. stomatis*–*P.micra* and *P. stomatis*–*P. micra*-*ETBF* showed higher AUCs than the single *P. stomatis* (*P_P.stomatis_*_–_ *_P.micra_* = 0.0411, *P_P.stomatis_*_–_ *_P.micra-ETBF_* = 0.0416) (**Figure 5D**). In terms of Youden index, the *P. stomatis*–*P. micra* marker was equal to the *P. stomatis*–*P. micra*–*ETBF* marker with 76.2% sensitivity and 80.8% specificity (**Table 1**).

### *P. stomatis* Were Positively Related to Adenoma Recurrence After Resection of LSTs

**Figure 6:**
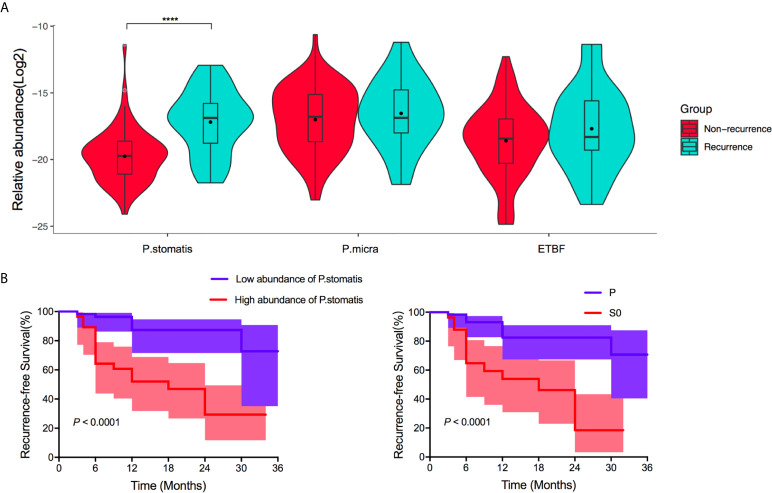
Performance of *ETBF, P. stomatis*, and *P. micra* on adenoma recurrence after resection of LST. **(A)** The fecal relative abundance of *P. stomatis, P. micra*, and *ETBF* among non-recurrence group and recurrence group. *P. stomatis* was related with adenoma recurrence. **(B)** Kaplan-Meier estimates for survival probability based on the abundance levels of *P. stomatis* in LST and histopathology of LST. High abundance of *P. stomatis* and S0 stage LST indicted adenoma recurrence. *P. stomatis, Peptostreptococcus stomatis; P. micra, Parvimonas micra; ETBF, Enterotoxigenic Bacteroides fragilis*; P, LSTs with hyperplastic type or llow-grade dysplasia; S0, LSTs with intramucosal carcinoma or high-grade dysplasia. ****P < 0.0001.

Considering the relationship between signature bacteria and tumoral development of LST, the next step of this study aims to find if there were differentiations in the above three bacteria composition between recurrence or non-recurrence adenoma after resection of LSTs. After 36 months’ follow-ups, there were 23 LST patients with adenoma recurrence among 92 LST patients. To this end, the distribution of *ETBF*, *P. stomatis*, and *P. micra* among the recurrence group and non-recurrence group was first evaluated. The recurrence group exhibited a predominant abundance of *P. stomatis* (*P* < 0.0001). Besides, no difference of the other two bacteria between the two groups were detected (**Figure 6A**) and *P. stomatis* presented strong value on predicting the adenoma recurrence (AUC 0.800, 95% CI 0.678–0.915, *P* < 0.0001). Based on these results, the enrolled patients were divided into high abundance of *P. stomatis* group and low abundance of *P. stomatis* group. Univariate analysis showed the dysplasia type of LST and the abundance of *P. stomatis* were related to the adenoma recurrence, while gender, age, history of hypertension or diabetes mellitus, diameter, location, topography, histopathology futiled (**Supplementary Table 5**). The survival curve showed the S0 LSTs (HR = 2.78, *P* = 0.026) and high-abundance of *P. stomatis* (HR = 3.88, *P* = 0.008) were related to the adenoma recurrence event (**Figure 6B**). Furthermore, the abundance difference of *P. stomatis* in early-recurrence group and late-recurrence group was also tested to be no significant difference between the above two groups (**Supplementary Figure 6**).

## Discussion

Growing evidence revealed that alteration in gut microbiome relates to colorectal neoplasms (12, 28, 29). However, LST, as a principal precursor lesion of CRC, was never reported about the intestinal microbiota signature. Moreover, early detection of LSTs and early prediction of the risk of adenoma recurrence after LST resection were essential to prevent CRC. Herein, the gut microbiota signature of LST cohort by means of 16S rRNA gene sequencing was delineated in this study. The results demonstrate that LST microbial dysbiosis was characterized by relative high abundance of the genus *Lactobacillus*-*Streptococcus* and the species *ETBF*-*P. stomatis*–*P. micra.* Based on the microbial signature, fecal microbial biomarkers *ETBF*–*P. stomatis*–*P. micra* were defined as early noninvasive biomarkers of LST. It indicates microbiome may form a synergistic system resulting diseases. Moreover, *P. stomatis* behaved high accuracy on predicting adenoma recurrence after LST resections.

*ETBF* has been known as a contributor to many colonic diseases including colonic dysfunctions, intestinal inflammation, oncogenic transformation and colorectal precancerous and cancerous lesions (23–27). Combined action of IL-17 and *ETBF* on colonic epithelial cells suppressed T cell proliferation and promoted the differentiation of monocytic-myeloid-derived suppressor cells (MO-MDSCs) (26). *Bacteroides fragilis*-associated lncRNA1(BFAL1) is an important modulator of *ETBF*-induced carcinogenesis. Thus, for *ETBF*-induced CRC, BFAL1 can be a potential therapeutic target (27). The *ETBF*-bearing biofilms in colon biopsies from CRC patients susceptibility loci were recently reported to strongly suggest that *ETBF* plays a key role in CRC development (30). *P.stomatis* and *P. micra* are both part of the oral and gut commensal microbioma and gram-positive anaerobic bacterium (31, 32). These organisms have been concerned as contributing agents of many diseases (12, 13, 31, 33–35), including oral squamous cell carcinoma (33) and apical abscess (34). A study of 526 fecal shotgun metagenome data sets revealed seven enriched and core bacteria in CRC (28). The core set of bacteria included: *ETBF*; four oral bacteria of *Fusobacterium nucleatum* (*F. nucleatum*) *(* 36, 37), *Parvimonas micra*, *Prevotella intermedia* (38), and *Porphyromonas asaccharolytica* (39); and two other bacteria, *Thermanaerovibrio acidaminovorans* (28) and *Alistipes finegoldii* (40). Yachida et al. (29) collected 616 stool samples and found these bacteria were observed in intramucosal carcinomas and multiple CRAs. Stage-specific analysis disclosed two elevated patterns of significant species: the first only increased in early stage, whereas the second one was elevated across early to later stages. The second pattern was characterized by *F. nucleatum, Solobacterium moorei* (38)*, P. stomatis, Peptostreptococcus anaerobius* (41, 42)*, Lactobacillus sanfranciscensis, P. micra*, and *Gemella morbillorum* (43). *ETBF*, *P. stomatis*, and *P. micra* were crucial to colorectal carcinogenesis while they were rarely reported to be biomarkers to detect precancerous lesions, especially in LSTs.

Many studies have applied the abundance of various bacteria to distinguish CRC patients from HCs. Among bacterial candidates, *F. nucleatum* appeared as a key marker either when being combined with other potential bacteria (13, 17, 44) or quantified alone (16, 21), specifically combined with *Clostridium symbiosum* (17). These findings provide sturdy support that a limited number of targeted bacterial markers may provide accurate stool-based noninvasive diagnostic value. In this study, the fecal abundance of the three bacteria *ETBF*–*P. stomatis*–*P. micra* displayed considerably high sensitivity and specificity in detecting LST, especially *P. stomatis*. While combinations of *P. stomatis*–*P. micra*–*ETBF* revealed higher diagnostic potential compared with the single biomarker *P. stomatis* and combined biomarkers of *P. stomatis*–*P. micra*, *P. stomatis*–*ETBF, P. micra*–*ETBF*. The wide application of the above bacterial markers in LST detection may be rather practical since qPCR detection of fecal bacterial DNA is apparently more cost-effective and reliable than strict endoscopic screening.

Adenoma recurrence after endoscopic resection of LSTs is frequent (7, 8, 45, 46). Despite the widespread use of endoscopic resection, few studies examined the risk factors of adenoma recurrence after standard therapy for LST. Cancerous LSTs were related with adenoma recurrence event after endoscopic therapy (47). Some studies showed that independent risk factors recurrent adenoma were lesion size >60 mm, lesion occupying 75% of the luminal circumference, high grade dysplasia of LSTs, adjunctive argon plasma coagulation and piecemeal resection (8, 47, 48). Furthermore, adenoma recurrence surveillance requests regular endoscopy screening. Many LST patients not developing adenoma recurrence may be subjected to ultimately unnecessary surveillance procedures. Inconvenient bowel preparation, well-trained technician and carefully installed devices before endoscopy are resource-consuming and could limit the use for population-wide screening. Based on the results of this study, it is worth noting that the relative high abundance of *P. stomatis* positively correlated with adenoma recurrence after LST resection. Therefore, application of the biomarker *P. stomatis* to predict adenoma recurrence was promising and feasible.

Furthermore, *ETBF*–*P. stomatis*–*P. micra* modulation, especially *P. stomatis* may be important for LST development, adenoma recurrence after LST resection and even colorectal carcinogenesis. It will require concerted efforts to translate it into a clinical product to find the appropriate interventional methods to manipulate the microbiota.

However, there is no totally clear answer about the role of *ETBF*–*P. stomatis*–*P. micra* in the development of LST or CRC. The process is intricate and influenced by environmental and genetic factors. Mechanisms include immune regulation, metabolism of dietary components, genotoxin production, and inflammation (49–51). There remained a question that what are the driver and passenger bacteria in LST development? As described, the three above bacteria increased IL-6 secretion in LST group. IL-6 is an inflammatory cytokine and plays important roles in microenviroment of colorectal cancers (52–55). IL-6 level was reported to be higher in CRC tissues compared with noncancerous tissues (52). Moreover, STAT3 activation through IL-6/IL-11 in cancer associated fibroblasts (CAFs) promoted CRC development and poor prognosis (53). IL-6 trans-signaling in a mouse model of CRC acted downstream of epidermal growth factor signaling in myeloid cells (54). It could be hypothesized that the three bacteria may induce inflammatory environment to accelerate the development of LSTs, while it deserves in-depth studies about this carcinogenesis process.

The strengths of the present study were as follows. First, we reported for the first time that the bacteria signature of LST. Second, we found fecal microbiome signature (*ETBF*–*P. stomatis*–*P. micra*) can effectively predict the occurrence of LSTs. Moreover, *ETBF*, *P. stomatis*, and *P. micra* were related to the S0 stage LST. Third, our study provided new evidence that *P. stomatis* exhibited the high accuracy on predicting the adenoma recurrence after endoscopic resection of LSTs.

Despite the clinical data for endoscopic resections was collected prospectively and all consecutive patients were enrolled, this study was limited by its retrospective design, as all results were only from the single tertiary center and could not be generalizable. In future, studies in large cohorts including need to be carried out to enroll more patients, which can derive the best diagnostic algorithm across populations.

## Conclusion

In conclusion, we observed, for the first time, fecal microbiome signature (*ETBF*-*P. stomatis*–*P. micra*) can effectively predict the presence of LSTs. Moreover, *ETBF*, *P. stomatis*, and *P. micra* were related to malignant LST and *P. stomatis* exhibited the high accuracy on predicting the adenoma recurrence after endoscopic resection of LSTs. Thus, the signature bacteria of LST can provide a noninvasive method to early detect LST and predict the adenoma recurrence risk after resections.

## Data Availability Statement

The 16S rRNA sequences were deposited at NCBI Sequence Read Archive repository with accession code SRP317984.

## Ethics Statement

The studies involving human participants were reviewed and approved by the Ethics Committee of Renji Hospital, School of Medicine, Shanghai Jiaotong University. The patients/participants provided their written informed consent to participate in this study.

## Author Contributions

XS analyzed the data and wrote the manuscript. XS, JLL, JQL, YZ, XL, and QG conceived the study and collected samples. YC and XC performed the pathological diagnosis. YXC and J-YF revised the manuscript. J-YF designed and supervised the study. All authors contributed to the article and approved the submitted version.

## Funding

This study was supported by grants from the National Natural Science Foundation of China (81421001, 81530072, 81830081).

## Conflict of Interest

The authors declare that the research was conducted in the absence of any commercial or financial relationships that could be construed as a potential conflict of interest.
