# Supplementary material for: Fecal Enterotoxigenic Bacteroides fragilis–Peptostreptococcus stomatis–Parvimonas micra Biomarker for Noninvasive Diagnosis and Prognosis of Colorectal Laterally Spreading Tumor
Source: Front Oncol. 2021 May 11;11:661048. doi: 10.3389/fonc.2021.661048 (PMC8144651; doi:10.3389/fonc.2021.661048)
Supplement: Supplementary file 1 [file DataSheet_1.docx]

**Supplementary Tables**

**Supplementary Table 1. Primer sequence of ETBF, P.stomatis, P.micra, L. johnsonii and 16s rRNA**

|  | **Forward sequence(5'-3')** | **Reverse sequence(5'-3')** |
| --- | --- | --- |
| **ETBF** | GGCGAACTCGGTTTATGCAG | AGGCAGTAAAGCCATCCAGC |
| **P. stomatis** | GCGAGGGTTTGCTCAGTATTG | TGATATATCTGCGATGCCGC |
| **P. micra** | ﻿GTCACTACGGAAGAATTTGTC | ﻿GGCTTGAGCGATAATAACTTC |
| **L. johnsonii** | TGGAAACAGGTGCTAATACCG | CAGTTACTACCTCTATCTTTCTTCACTAC |
| **16S rRNA** | GGTGAATACGTTCCCGG | TACGGCTACCTTGTTACGACTT |

**Supplementary Table 2. Basic democratic information for HC-CRA-LST-CRC cohorts**

|  | **N** | | **Age** | **M/F** |
| --- | --- | --- | --- | --- |
| **HC group** | | 113 | 63.1 | 1.2 |
| **CRA group** | | 208 | 63.8 | 1.7 |
| **LST group** | | 109 | 63.9 | 1.2 |
| **CRC group** | | 45 | 65.0 | 1.4 |

**Supplementary Table 3. The relevant bacteria co-occurred with Bacteroides fragilis in colorectal neoplasms**

| **Spiecies** | **Cor *** | **p value*** | **Cor **** | **p value**** | **OR** | **p-value***** | **95% CI of OR** | |
| --- | --- | --- | --- | --- | --- | --- | --- | --- |
| **Peptostreptococcus.stomatis** | 0.1768 | 0.0000 | 0.1862 | 0.0000 | 15.4903 | 0.0000 | 7.9800 | 33.6377 |
| **Parvimonas.micra** | 0.1818 | 0.0000 | 0.1227 | 0.0034 | 12.3751 | 0.0000 | 6.1172 | 28.3540 |
| **Bilophila.wadsworthia** | 0.1615 | 0.0001 | -0.2805 | 0.0000 | 2.1916 | 0.0001 | 1.4685 | 3.2997 |
| **Clostridium.sphenoides** | 0.4269 | 0.0000 | -0.0816 | 0.0498 | 2.0226 | 0.0187 | 1.0878 | 3.8484 |
| **Bacteroides.intestinalis** | -0.0924 | 0.0193 | -0.3271 | 0.0000 | 1.8365 | 0.0001 | 1.3313 | 2.5410 |
| **Ruminococcus.sp** | -0.1247 | 0.0019 | -0.2905 | 0.0000 | 1.8242 | 0.0009 | 1.2633 | 2.6462 |
| **Bacteroides.vulgatus** | -0.0752 | 0.0122 | -0.1173 | 0.0001 | 1.7092 | 0.0006 | 1.2499 | 2.3515 |
| **Alistipes.onderdonkii** | -0.0839 | 0.0140 | -0.3090 | 0.0000 | 1.6093 | 0.0000 | 1.2785 | 2.0273 |
| **Barnesiella.viscericola** | -0.0824 | 0.0366 | -0.4222 | 0.0000 | 1.6063 | 0.0031 | 1.1619 | 2.2250 |
| **Eubacterium.eligens** | -0.1253 | 0.0003 | -0.3700 | 0.0000 | 1.5579 | 0.0001 | 1.2374 | 1.9629 |
| **Eubacterium.desmolans** | -0.1152 | 0.0032 | -0.4135 | 0.0000 | 1.5005 | 0.0103 | 1.0957 | 2.0576 |
| **Butyricimonas.virosa** | -0.0864 | 0.0281 | -0.4974 | 0.0000 | 1.4170 | 0.0354 | 1.0208 | 1.9687 |
| **Methanobrevibacter.smithii** | -0.0988 | 0.0098 | -0.3595 | 0.0000 | 1.4137 | 0.0160 | 1.0621 | 1.8827 |
| **Bacteroides.xylanisolvens** | -0.0837 | 0.0154 | -0.4151 | 0.0000 | 1.4094 | 0.0029 | 1.1189 | 1.7761 |
| **Alistipes.finegoldii** | -0.0939 | 0.0115 | -0.4396 | 0.0000 | 1.4086 | 0.0084 | 1.0874 | 1.8255 |
| **Alistipes.putredinis** | -0.1346 | 0.0000 | -0.2492 | 0.0000 | 1.4033 | 0.0051 | 1.1008 | 1.7913 |
| **Dialister.invisus** | -0.1507 | 0.0000 | -0.4271 | 0.0000 | 1.3937 | 0.0078 | 1.0860 | 1.7889 |
| **Bacteroides.cellulosilyticus** | -0.0754 | 0.0358 | -0.3521 | 0.0000 | 1.3914 | 0.0060 | 1.0935 | 1.7710 |
| **Oscillibacter.valericigenes** | -0.1189 | 0.0011 | -0.4657 | 0.0000 | 1.3101 | 0.0301 | 1.0206 | 1.6821 |

*: Pearson’s correlation coefficient; **: Spearman’s correlation coefficient; ***: Odds ratio(OR)

**Supplementary Table 4. The abundance of IL-6, TNF-α, IL-1β, IL-8, IL-10 of 35 LST patients obtained from clinical laboratory test**

| **Patient ID** | **Relative abundance of Ps(log2)** | **Relative abundance of Pm(log2)** | **Relative abundance of ETBF(log2)** | **IL-6(pg/ml)** | **TNF-α(pg/ml)** | **IL-1β(pg/ml)** | **IL-8(pg/ml)** | **IL-10(pg/ml)** |
| --- | --- | --- | --- | --- | --- | --- | --- | --- |
| **1** | -14.03255348 | -11.21054459 | -11.34625013 | 12.5 | 13.54 | 15.99 | 292.8 | 71.18 |
| **2** | -15.98615341 | -15.22338441 | -12.28118515 | 2.48 | 11.26 | 15.35 | 252.9 | 69.02 |
| **3** | -16.63444405 | -17.01571083 | -16.70009327 | 3.89 | 12.32 | 6.22 | 104 | 67.13 |
| **4** | -16.63439904 | -17.01750138 | -15.87160492 | 3.31 | 12.4 | 5.66 | 165.9 | 56.96 |
| **5** | -16.94039993 | -15.14039993 | -18.75093746 | 3.53 | 12.72 | 16.21 | 153.5 | 44.84 |
| **6** | -16.95192604 | -15.97075081 | -17.25189018 | 6.03 | 13.41 | 14.03 | 151 | 53.7 |
| **7** | -17.02338441 | -13.19367981 | -11.36452103 | 11.9 | 11 | 16.5 | 110 | 51.3 |
| **8** | -17.0782402 | -16.89276505 | -21.57323074 | 3.43 | 11.39 | 9.08 | 216.2 | 54.44 |
| **9** | -17.2240368 | -15.4240368 | -17.28040123 | 4.45 | 13 | 16.45 | 156 | 50.84 |
| **10** | -17.41600685 | -17.73193359 | -19.85241795 | 2.07 | 12.96 | 10.9 | 66.5 | 47.56 |
| **11** | -17.47663383 | -19.2242012 | -16.99384499 | 6.54 | 10.6 | 17.22 | 230 | 66.35 |
| **12** | -17.70158367 | -15.72648144 | -17.45370388 | 2.96 | 11.64 | 6.79 | 226.2 | 60.73 |
| **13** | -18.08635597 | -13.58017349 | -23.36733627 | 2.77 | 9.12 | 5.58 | 94.6 | 59 |
| **14** | -18.24429604 | -16.44429604 | -18.85648727 | 3.78 | 10.67 | 15.27 | 180 | 64.91 |
| **15** | -18.51266971 | -16.71266971 | -17.18367577 | 3.71 | 11.22 | 13.07 | 123.5 | 51.37 |
| **16** | -18.51416473 | -15.14305115 | -13.73918152 | 8.51 | 11.1 | 10.2 | 196 | 56.48 |
| **17** | -18.60078697 | -14.03778839 | -20.71509552 | 2.01 | 10.71 | 7.16 | 299.9 | 53.82 |
| **18** | -18.61973934 | -18.6588316 | -17.54313469 | 3.87 | 9.12 | 7.5 | 240 | 52.21 |
| **19** | -19.03487091 | -14.64858627 | -19.12150383 | 2.84 | 9.71 | 17.85 | 156 | 44 |
| **20** | -19.07251196 | -17.27251196 | -19.17943764 | 2.34 | 9.61 | 6.68 | 154.1 | 38 |
| **21** | -19.23229866 | -14.37247849 | -22.87849808 | 5.32 | 9.96 | 6.89 | 195 | 58.19 |
| **22** | -19.39134299 | -17.59134299 | -17.32711697 | 7.47 | 11.9 | 5.93 | 89.9 | 66.22 |
| **23** | -19.39308052 | -16.2603302 | -20.16225815 | 4.19 | 13.55 | 15.21 | 66.1 | 69.24 |
| **24** | -19.47140007 | -16.42363167 | -16.95917511 | 8.76 | 8.83 | 14.2 | 142 | 52.94 |
| **25** | -19.47801666 | -14.06429863 | -15.63506317 | 3.83 | 10.37 | 15.36 | 150 | 69.51 |
| **26** | -19.70061188 | -16.8054924 | -21.09740448 | 2.72 | 13.55 | 17.98 | 181.2 | 51 |
| **27** | -19.82380562 | -16.37737274 | -21.66481304 | 2.92 | 10.06 | 7.98 | 174.6 | 66.97 |
| **28** | -19.8965004 | -20.2700367 | -20.29323196 | 2.92 | 12.15 | 11.57 | 247.5 | 58.35 |
| **29** | -20.15590267 | -16.01349354 | -20.60093021 | 3.41 | 12.66 | 7.97 | 180.8 | 60.91 |
| **30** | -20.1874588 | -15.20771408 | -18.06353855 | 2.25 | 11.93 | 17.35 | 272.6 | 53 |
| **31** | -20.33088474 | -18.78739262 | -16.61048031 | 2.47 | 11.79 | 6.43 | 298.4 | 54 |
| **32** | -21.00015621 | -16.79081059 | -20.06392574 | 4.03 | 9.73 | 16.88 | 88 | 55.93 |
| **33** | -21.09799652 | -14.27173996 | -24.85114098 | 3.78 | 11.56 | 12.42 | 243 | 61 |
| **34** | -21.13161449 | -18.36994839 | -18.39596558 | 3.04 | 9.86 | 7.39 | 98.6 | 36.85 |
| **35** | -21.66392403 | -19.80779839 | -19.0898838 | 2.55 | 11.98 | 7.64 | 255.5 | 58 |

**Supplementary Table 5. Univariate analysis about the risk factors related with adenoma recurrence after LST resection**

|  | | **Non-recurrence** | **Recurrence** | **P-value** |
| --- | --- | --- | --- | --- |
| **Gender** |  | |  | 0.481 |
| **Male** | 39 | | 11 |  |
| **Female** | 30 | | 12 |  |
| **Age(year)** |  | |  | 0.163 |
| **<45** | 3 | | 3 |  |
| **≥45** | 66 | | 20 |  |
| **History of HTN** |  | |  | 0.156 |
| **Yes** | 27 | | 13 |  |
| **No** | 42 | | 10 |  |
| **History of DM** |  | |  | 0.640 |
| **Yes** | 32 | | 12 |  |
| **No** | 37 | | 11 |  |
| **Size(cm)** |  | |  | 0.475 |
| **≤2** | 27 | | 11 |  |
| **>2** | 42 | | 12 |  |
| **Topography** |  | |  | 0.470 |
| **G** | 38 | | 15 |  |
| **NG** | 31 | | 8 |  |
| **Location** |  | |  | 0.065 |
| **Proximal colon** | 52 | | 12 |  |
| **Distal colon** | 17 | | 11 |  |
| **Histopathology** |  | |  | 0.574 |
| **Tranditional adenoma** | 51 | | 19 |  |
| **Serrated polyp** | 18 | | 4 |  |
| **Dysplasia type** |  | |  | 0.002 |
| **P** | 53 | | 9 |  |
| **S0** | 16 | | 14 |  |
| **Abundance of P.stomatis** |  | |  | < 0.0001 |
| **Low** | 57 | | 6 |  |
| **High** | 12 | | 17 |  |
