# Supplementary material for: Fecal Enterotoxigenic Bacteroides fragilis–Peptostreptococcus stomatis–Parvimonas micra Biomarker for Noninvasive Diagnosis and Prognosis of Colorectal Laterally Spreading Tumor
Source: Front Oncol. 2021 May 11;11:661048. doi: 10.3389/fonc.2021.661048 (PMC8144651; doi:10.3389/fonc.2021.661048)
Supplement: Supplementary file 2 [file DataSheet_2.docx]

**Supplementary Figure Legend**

**Figure S1** The Core and Rarefaction curve of 16s rRNA sequencing samples. **(A)** The Core curve of 16s rRNA sequencing samples on OTU level. **(B)** The Rarefaction curve of 16s rRNA sequencing samples on OTU level.

**Figure S2** Community barplot analysis on genus level of all samples in the three groups. HC=healthy control. CRA=colorectal adenoma. LST=laterally spreading tumor. CRC=colorectal carcinoma.

**Figure S3** The fecal relative abundance distribution of *ETBF*, *P. stomatis* and *P. micra.* **(A)** LSTs located in proximal colon group and LSTs located in distal colon group. **(B)** LST-G group and LST-NG group. **(C)** LSTs with traditional adenoma group and serrated polyps group. *ETBF*= *Enterotoxigenic Bacteroides fragilis P. stomatis*= *Peptostreptococcus stomatis*. *P. micra= Parvimonas micra.* proximal = caecum, ascending colon, hepatic flexure, transverse colon or splenic flexure. distal =descending colon, sigmoid colon and rectum.

**Figure S4** The expression of TNF-α, IL-1β, IL-8, IL-10 with the abundance of the three bacteria. **(A)** The expression of TNF-α, IL-1β, IL-8, IL-10 with the abundance of *Ps.* **(B)** The expression of TNF-α, IL-1β, IL-8, IL-10 with the abundance of *Pm.* **(C)** The expression of TNF-α, IL-1β, IL-8, IL-10 with the abundance of *ETBF. Ps*= *Peptostreptococcus stomatis*. *Pm=Parvimonas micra. ETBF*=*Enterotoxigenic Bacteroides fragilis.*

**Figure S5** The fecal relative abundance of *Lactobacillus johnsonil* among groups. HC=healthy control. CRA=colorectal adenoma. LST=laterally spreading tumor. CRC=colorectal carcinoma*.*

**Figure S6** The fecal relative abundance of *P. stomatis* among early-recurrence LST group and late-recurrence LST group. *P. stomatis*= *Peptostreptococcus stomatis*.
