# Supplementary figures and images for: Fecal Enterotoxigenic Bacteroides fragilis–Peptostreptococcus stomatis–Parvimonas micra Biomarker for Noninvasive Diagnosis and Prognosis of Colorectal Laterally Spreading Tumor
Source: Front Oncol. 2021 May 11;11:661048. doi: 10.3389/fonc.2021.661048 (PMC8144651; doi:10.3389/fonc.2021.661048)

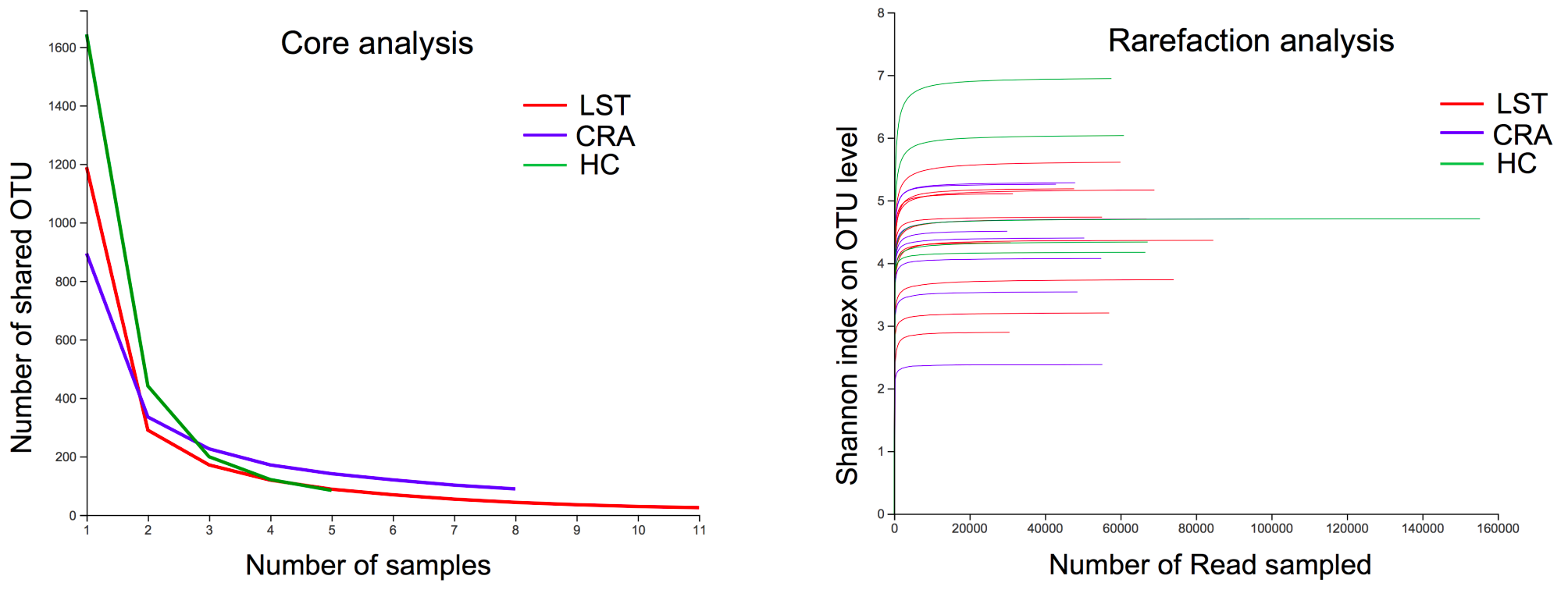

Supplement: Supplementary file 3 [file Image_1.tiff]

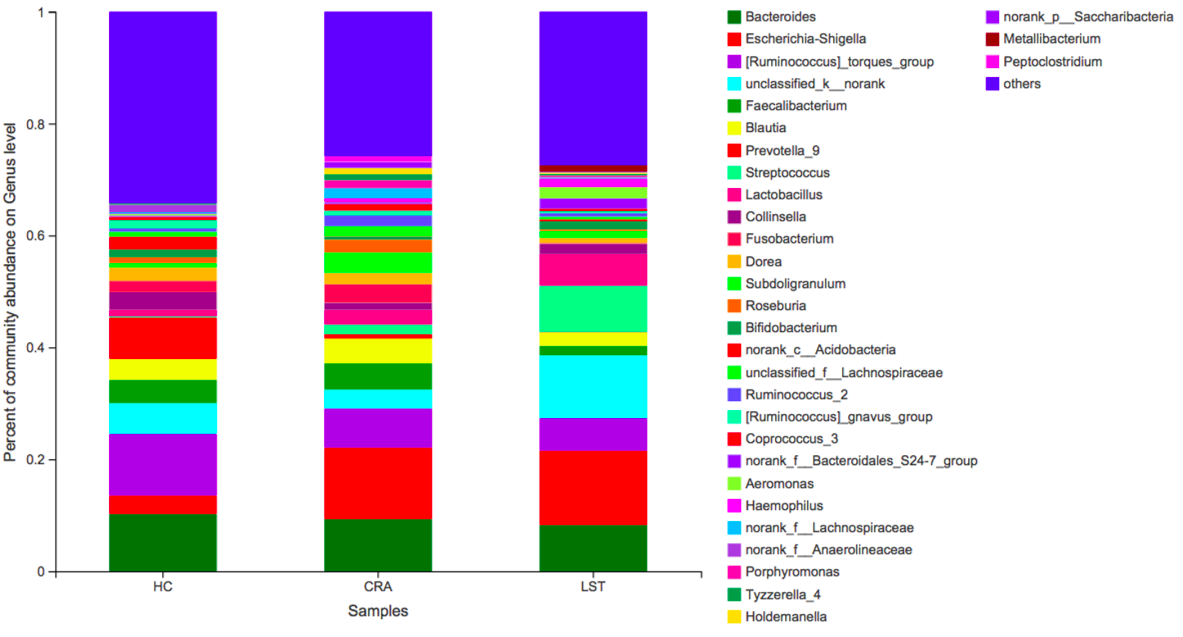

Supplement: Supplementary file 4 [file Image_2.tiff]

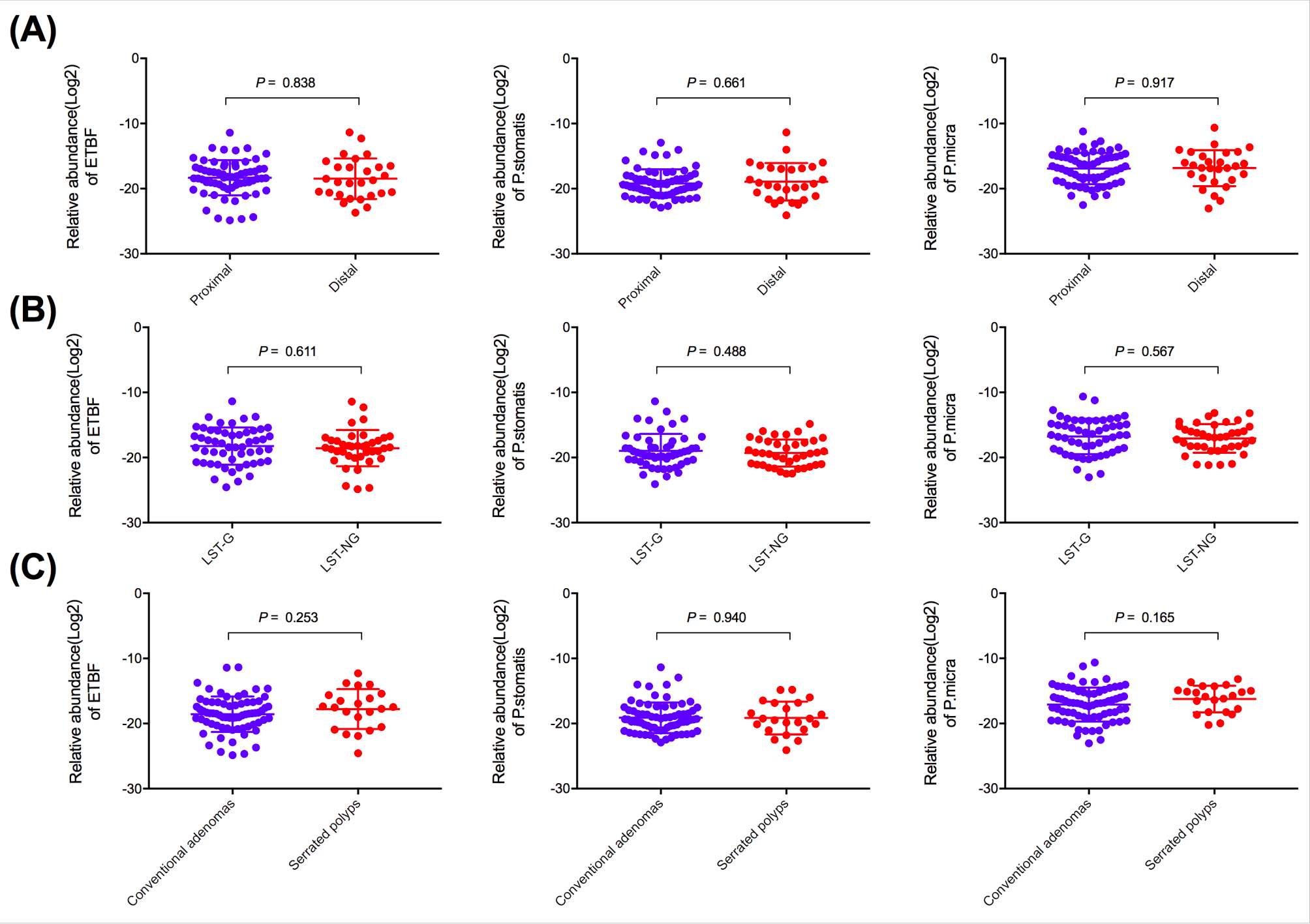

Supplement: Supplementary file 5 [file Image_3.tiff]

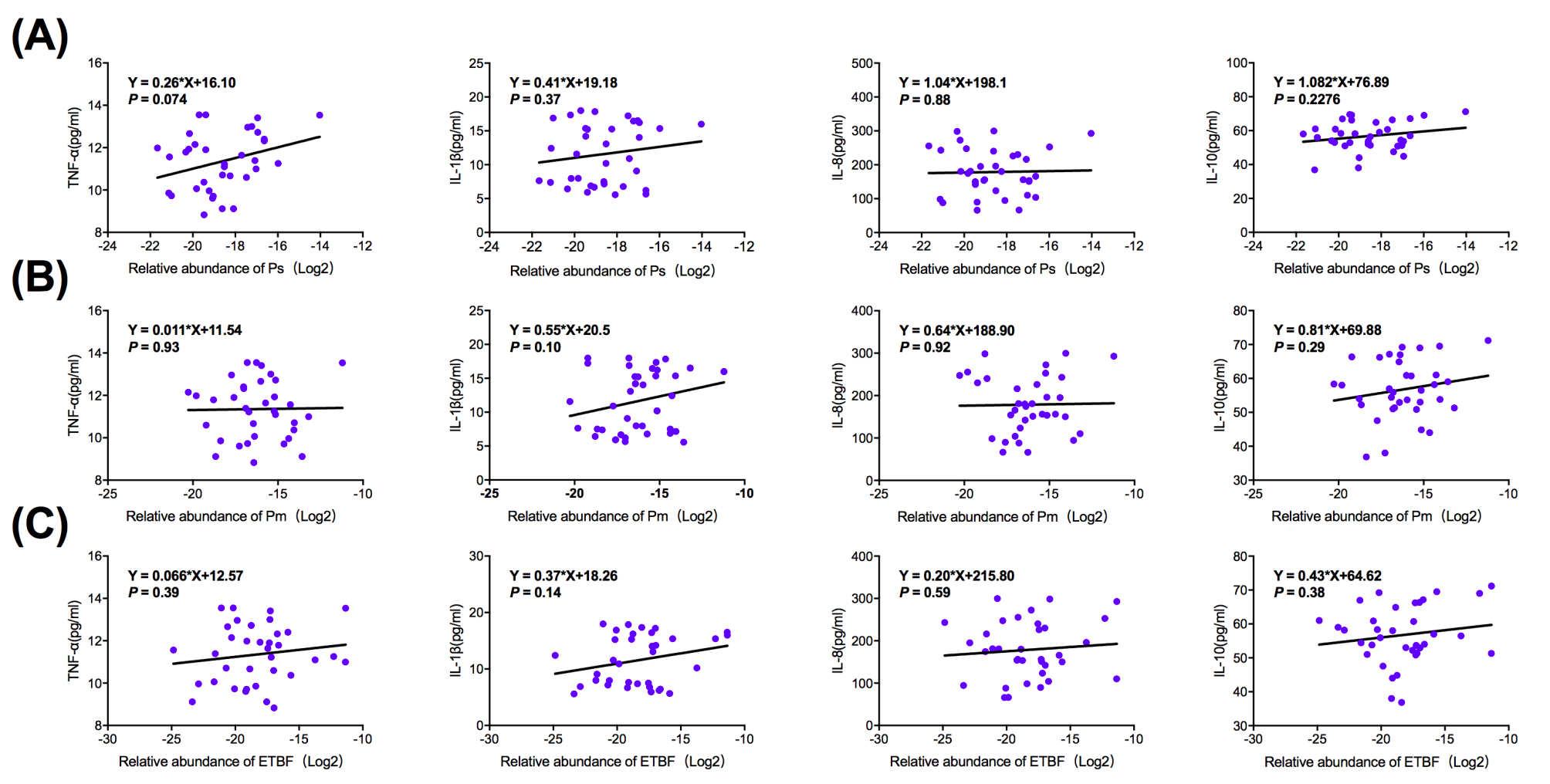

Supplement: Supplementary file 6 [file Image_4.tiff]

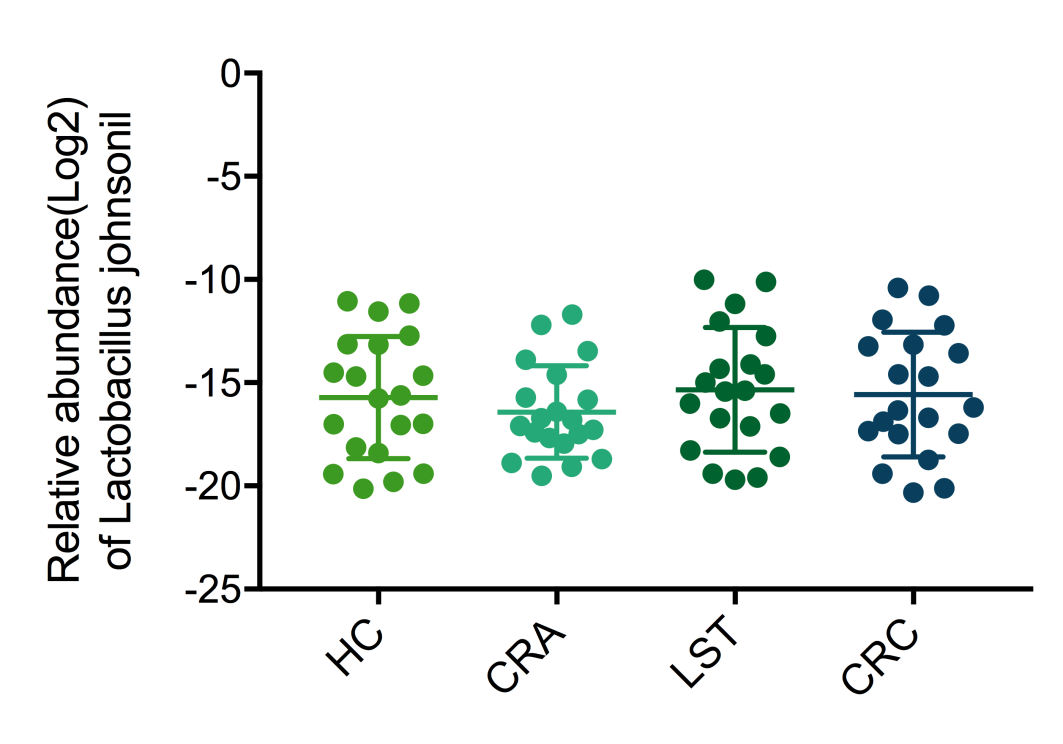

Supplement: Supplementary file 7 [file Image_5.tiff]

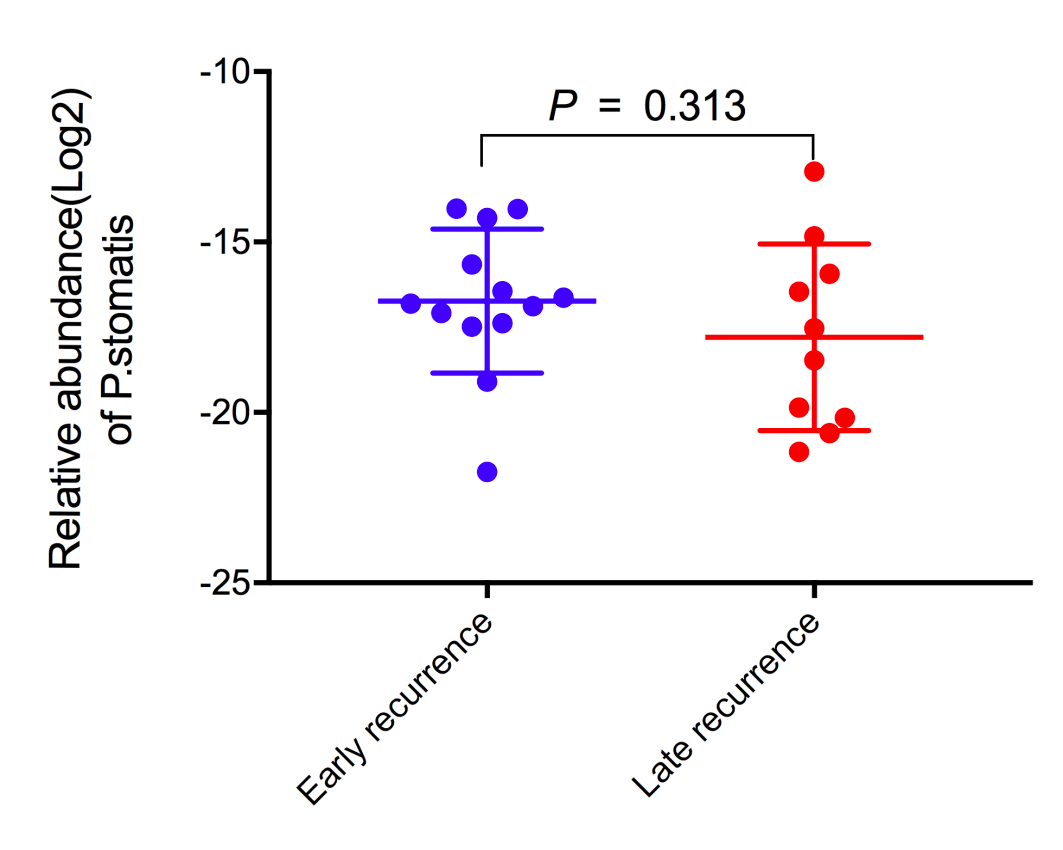

Supplement: Supplementary file 8 [file Image_6.tiff]
